# *Chlamydia trachomatis* Pgp3 Antibody Persists and Correlates with Self-Reported Infection and Behavioural Risks in a Blinded Cohort Study

**DOI:** 10.1371/journal.pone.0151497

**Published:** 2016-03-14

**Authors:** Patrick J. Horner, Gillian S. Wills, Antoinette Righarts, Sueli Vieira, Daphne Kounali, Dhanraj Samuel, Alan Winston, David Muir, Nigel P. Dickson, Myra O. McClure

**Affiliations:** 1 Jefferiss Trust Laboratories, Wright-Fleming Institute, Imperial College London, London, United Kingdom; 2 School of Social and Community Medicine, University of Bristol, Bristol, United Kingdom; 3 Department of Preventive and Social Medicine, University of Otago, Dunedin, New Zealand; 4 National Institute for Health Research Health Protection Research Unit (NIHR HPRU) in Evaluation of Interventions in partnership with Public Health England, University of Bristol, Bristol, United Kingdom; 5 Public Health for England, Colindale, London, United Kingdom; 6 Department of Infection and Immunity, Imperial College Healthcare Trust, London, United Kingdom; University of California, San Francisco, University of California, Berkeley, and the Children's Hospital Oakland Research Institute, UNITED STATES

## Abstract

*Chlamydia trachomatis* (Ct) serological studies in populations could help monitor changes in lifetime cumulative risk of infection. We developed a double-antigen sandwich ELISA based on the Ct-specific Pgp3 antigen, then tested blind stored sera from over 800 participants in a New Zealand birth cohort from Dunedin at ages 26, 32 and 38. The double-antigen sandwich ELISA was more sensitive than our previously characterised indirect Pgp3 ELISA. Pgp3 antibody was detected more often in women compared to men and correlated with increasing numbers of sexual partners, self-reported Ct, and younger age at sexual debut in both women and men. At age 26, 24.1% (99/411) of women were Pgp3 seropositive, as were 79.5% (35/44) of those reporting Ct infection; Pgp3 antibody persisted to age 38 in 96.5% (83/86). In men at age 26, the figures were 10.7% (47/442) and 25.0% (6/24), respectively, with high (83.9%) antibody persistence to age 38. At age 38, among those Pgp3 seropositive, 63.3% of women and 83.1% of men had not reported Ct infection. Thus, Ct-specific Pgp3 antibody was detected in most women reporting Ct infection and correlated with risk of infection in those who did not, with most infections remaining undetected. As this antibody persisted for at least twelve years in 96% of these women, serology could be used to evaluate Ct prevention programmes among women.

## Introduction

*Chlamydia trachomatis* (Ct) infection, if untreated in women, can result in pelvic inflammatory disease, a condition leading to significant reproductive morbidity [1–3]. Opportunistic or screening programmes have been recommended or implemented in several countries to reduce prevalence and, subsequently, incidence and reproductive sequelae [4, 5], but their effectiveness has never been confirmed by randomised controlled trials. While findings from the United Kingdom (UK) screening programme (aimed at all those under 25 years) provide a measure of current prevalence of those tested, a declining cumulative risk of infection would be a better marker of success [6–8].

We previously produced an indirect lgG Enzyme Linked Immunosorbent Assay (ELISA) to detect antibody to Ct-specific Pgp3 protein [9]. The Pgp3 protein is transcribed from the highly conserved Ct plasmid [10] that is not found in human *C*. *pneumoniae* isolates [11]. Pgp3 is also highly immunogenic in its native, trimeric form [12, 13] and antibody to Pgp3 does not cross react with *C*. *pneumoniae* proteins with which Ct shares many similar genes [8]. We have demonstrated the Pgp3 indirect ELISA is significantly more sensitive in detecting past Ct infection than three of the most commonly used ELISAs [9]. We refer to sensitivity as the proportion of individuals with past infection identified as positive by the assay i.e diagnostic sensitivity [14]. To maximise detection rates of past infection, we have developed a Pgp3 double-antigen sandwich ELISA, as has been done for other infections, including HIV-1, Hepatitis B and Hepatitis E [15–17]. Double-antigen assays require that each protein-specific antibody recognises the specific epitope of the antigen bound to an ELISA plate, as well as binding the same epitope on labelled Pgp3, allowing detection of lower antibody titres [18].

In this report we describe the new ELISA, and demonstrate its enhanced performance over other assays using the same samples that were originally used to validate our indirect assay [9]. We then examined Pgp3 antibody associations in participants of the New Zealand Dunedin Multidisciplinary Health and Development Study (DMHDS), a birth cohort study in which detailed information on sexual behaviour and health have been collected at regular intervals from age 18–38 years [19]. Stored sera collected at ages 26, 32 and 38 years were tested by our double-antigen ELISA, the findings compared with self-reported Ct infection and sexual behaviour, and the persistence of the antibody response measured over this 12-year period were determined.

## Methods

### Ct-positive and -negative control serum samples

Serum samples previously used to characterize our indirect ELISA [9] were available from 342 patients (including 182 men, 158 women and two of unknown sex) attending the Milne Centre, Bristol and the Jefferiss Wing, London Genitourinary Medicine (GUM) clinics. All patients had been diagnosed as Ct organism-positive at least one month previously.

The negative control sera were from 505 children aged between two and 13 years held at the Department of Diagnostic Virology, Imperial College London. These children were assumed to be Ct unexposed [9]. Ethical approval for the study was given by the South West—Central Bristol Research Ethics Committee [05/Q2003/48].

### Pgp3 double-antigen sandwich ELISA

Biotin-labelled Pgp3 was produced using the EZ-Link Sulfo-NHS-Biotinylation Kit (Thermo Scientific). Optimised assay conditions were determined by checkerboard titrations, as previously described [9]. Maxisorp microtitration plates (Nunc) were coated with unlabelled Pgp3 with bovine serum albumin (BSA) in carbonate buffer, pH 9.6 (Sigma) at 4°C. The protein-coated wells were blocked and stabilised by dilution buffer (PBS with 0.05% Tween-20 (PBST) (Sigma) with 1% Hammersten casein (GE Healthcare)) containing 5% sucrose (Sigma). Bound protein was incubated with either Pgp3 antibody-positive or negative defibrinated plasma [20] (25μl) diluted in dilution buffer (75μl) containing BSA at 37°C. After washing with PBST, biotinylated Pgp3 was added, incubated at 4°C, washed and incubated with horseradish peroxidase (HRP)-labelled streptavidin (Thermo Scientific). Finally, HRP activity was measured with TMB substrate (Biorad).

Assay cut-off was determined by receiver operating characteristic (ROC) analysis of absorbance (450–620nm) values on 505 paediatric samples and 342 samples from GUM patients, previously used to characterize our indirect ELISA [9]. As before, we determined specificity on the 494 samples from micro-immunofluorescence (MIF) assay-negative children [9].

### Other Ct serology assays

The double-antigen assay performance was compared to our indirect IgG ELISA which takes less operator time and less sample volume (1μl) [9], and to the Ct-IgG-pELISA plus Medac assay (Medac, Wedel, Germany) [3, 9, 21, 22], the SeroCT-IgG ELISA (Savyon Diagnostics, Ashdod, Israel) [3, 9, 22], the Ct IgG EIA (Ani Labsystems, Vantaa, Finland) [3, 7, 9, 22, 23] and Ct antibody at 1 in 32 titre using the *C*. *pneumonia* IgG/IgM MIF test kit (Ani Labsystems, Finland) [3, 9].

### Epidemiological sample

This DMHDS was formed of 1037 children (539 males and 498 females) born in Dunedin, New Zealand between April 1972 and March 1973, who participated in the first assessment at the age of three [19]. Sexual health and behaviour information was collected at ages 18, 21, 26, 32 and 38, and sera were available from the last three assessments.

Information on sexual behaviour and sexually transmitted infections (STIs) was collected by computerised questionnaire. Those reporting sexual intercourse were asked at age 21 about ever having had an STI, and at ages 26, 32 and 38 if they had one since the previous assessment. If an STI diagnosis was reported, the participant confirmed the specific infection, which was assumed to be treated. At each assessment the participants were asked how many sexual partners they had ever had. The total number of opposite and same-sex partners were combined at each assessment and grouped as 0, 1, 2–4, 5–9, 10–19 and 20 or more. Age at first intercourse was based on the first reported experience of vaginal intercourse or same-sex contact reported at age 21. If none were reported by age 21, information given at age 38, when questions on first heterosexual intercourse were repeated, was used [24].

Of the female cohort survivors, 83.2% (411/494) completed questionnaires on sexual behaviour and provided serum at the age 26 assessment, as did 87.8% (432/492) at the age 32 and 91.4% (448/490) at the age 38 assessments. Of the male survivors, 84.0% (441/525) at age 26, 85.9% (449/523) at age 32 and 87.0% (450/517) at age 38 did so. The sera were assayed for Ct Pgp3 antibody by both the indirect and the double-antigen assays.

Ethical approvals for the early phases of the study were given by the Otago and the Southern Regional Ethics Committee as relevant for each stage of the study and for the Phase (age) 38 by the Lower South Regional Ethics Committee of the Ministry of Health [LRS/10/03/012]. Written informed consent was obtained from the members of the DMHDS cohort.

### Data analysis

Data were analysed using STATA 13 statistical software. ROC analyses were carried out comparing results from the six different assays. Comparison of the Areas Under the Curves (AUC) for the different assays followed procedures described in DeLong *et al*. [25]. These data were programmed in Stata statistical software through the commands roccomp. P-values were adjusted for multiple comparisons of AUCs using the Sidak method [26].

Population averaged generalised linear models for correlated binomial data with the probit link were used to model the probability of a positive test result for a Ct-exposed patient (sensitivity) according to gender for the different assays [27]. The same models were used for the probabilities of negative test results for paediatric samples (specificity).

Pairwise comparisons were carried out as post-estimation calculations based on these models with 95% confidence intervals (CI) adjusted for multiple comparisons using the Scheffe method [28].

Data from the Dunedin Study were analysed for the association of Ct Pgp3 antibody positivity with self-reported Ct and behaviour. Fisher's exact tests probed differences in proportions of those testing Pgp3 antibody positive. In the supplementary analysis (Table D in S1 text), testing by both the indirect and double-antigen ELISA was assessed and all who provided serum were included.

A *P*-value of <0.05 was considered significant for all statistical tests.

## Results

### Pgp3 double-antigen ELISA

By ROC analysis, the Pgp3 double-antigen ELISA gives a significantly higher AUC than all other assays assessed (p<0.0001–0.0002) (Table 1), facilitating discrimination between positive and negative sera. This is confirmed by pairwise comparisons of sensitivities of the assays (Table 2 and Tables A and B in S1 Text). A marked gender difference in the sensitivity of the double-antigen ELISA was observed. Of the 158 GUM clinic samples from women, 131 (82.9% [95% CI 77.0–88.8%]) were Ct-antibody positive (Table 2). This constitutes a 15.9% increase in sensitivity over the indirect ELISA (71.5% [95% CI 64.5–78.6%]). In men, sensitivity was 54.4% (95% CI 47.2–61.6%) (Table 2), a 15.0% increase over the indirect assay. In the 69 men with only one episode of Ct and no history of urethritis the sensitivity was 42.6% (95% CI 31.6–54.5%).

**Table 1:** Comparison of ROC analysis AUCs across the different assays.

Assay	No. of samples^a^	ROC Area Mean (95% CI)	Pr>chi2	Pr>chi2 (Sidak adjusted)
Pgp3 double antigen	846	0.819 (0.793–0.845)		
Pgp3 indirect	846	0.774 (0.746–0.801)	<0.0001	<0.0001
Anilabsystems	846	0.693 (0.665–0.721)	<0.0001	<0.0001
SeroCT	846	0.718 (0.690–0.745)	<0.0001	<0.0001
Medac	846	0.739 (0.712–0.766)	<0.0001	<0.0001
MIF	846	0.766 (0.739–0.793)	0.0002	0.0011

^a^The 505 paediatric and 341 GUM clinic samples were included in this analysis. One sample not tested by the Anilabsystems assay was not included.

**Table 2:** Sensitivity of the Ct antibody assays according to gender.

Assay	Positive samples/Total no. of samples	No. of samples with discordant results		%Sensitivity (95% CI)
		^a^Pgp3 double antigen -ve Assay +ve	^b^Pgp3 double antigen +ve Assay -ve	
**Female**				
Pgp3 double antigen	131/158	-	-	82.9 (77.0–88.8)
Pgp3 indirect	113/158	0	18	71.5 (64.5–78.6)
Anilabsystems	94/158	6	43	59.5 (51.8–67.1)
SeroCT	87/158	3	47	55.1 (47.3–62.8)
Medac	73/158	5	63	46.2 (38.4–54.0)
MIF	101/158	5	35	63.9 (56.4–71.4)
**Male**				
Pgp3 double antigen	99/182	-	-	54.4 (47.2–61.6)
Pgp3 indirect	86/182	6	19	47.3 (40.0–54.5)
Anilabsystems	73/181	19	45	40.2 (33.1–47.4)
SeroCT	72/182	20	47	39.6 (32.5–46.7)
Medac	77/182	27	49	42.3 (35.1–49.5)
MIF	88/182	21	32	48.4 (41.1–55.6)

^a^The numbers of samples giving negative results by the Pgp3 double antigen ELISA but positive results by each of the other assays.

^b^The numbers of samples giving positive results by the Pgp3 double antigen ELISA but negative results by each of the other assays.

Specificity of the double-antigen ELISA, determined on 494 Ct-negative paediatric sera, was 97.8% (95% CI 96.5–99.1%), the same as that for the indirect assay 97.8% (95% CI 96.5–99.1%), and comparable with the commercial Major Outer Membrane Protein (MOMP) assays (95% CI 94.7–99.0%) (Table 3 and Table C in S1 text).

**Table 3:** Specificities of the Ct antibody assays using true negative Ct control sera^a^

	Negative samples/Total no. of samples	Specificity (95% CI)
Pgp3 double antigen	483/494	97.8 (96.5–99.1)
Pgp3 indirect	483/494	97.8 (96.5–99.1)
Anilabsystems	489/494	99.0 (98.1–99.9)
SeroCT	479/494	97.0 (95.5–98.5)
Medac	468/494	94.7 (92.8–96.7)
MIF	-	-

^a^Specificities were determined excluding the 11 paediatric samples positive by MIF assay.

### Ct-specific Pgp3 antibodies in Dunedin Study samples

All Dunedin samples (n = 2641), including 10 for which there was no corresponding behavioural information, were assayed by the double-antigen and, for direct comparison, the indirect ELISAs (Table D in S1 Text). Of the 2365 samples that tested negative by the indirect, 243 were positive by the double-sandwich ELISA, consistent with the higher sensitivity of the new assay. Of the samples that tested positive by the indirect (n = 276), 260 (94.2%) remained positive by the double-antigen ELISA. Of the 16 discordant samples, 11 (from six individuals) were positive when assayed on an indirect ELISA without antigen present, hence those must have been false-positives. The remaining five samples (from three individuals) originally positive were negative by the second assay. This remains unexplained, although non-specific binding due to heterophilic antibodies and rheumatoid factor is a common occurrence in serological assays [29]. The double-antigen ELISA detects nearly double (1.75 times) the number of self-reported Ct cases compared to the indirect ELISA. Hence, only results from the double-antigen ELISA were used for the following analysis.

At all ages, more women than men were Pgp3 antibody positive (Tables 4 and 5). The proportion of seropositive women and men were, respectively, 24.1% and 10.7% (p<0.001) at age 26, 26.2% and 14.0% (p<0.001) at age 32, and 26.8% and 13.1% (p<0.001) at age 38. Among women who self-reported Ct, the percentages seropositive at ages 26, 32 and 38 were 79.5%, 75.0% and 74.6%, respectively; all significantly (p<0.001) higher than those who had never reported infection (Table 4). Among men self-reporting Ct by these ages, 25.0%, 33.3% and 27.0% were seropositive, respectively, again significantly higher than those who had undeclared infection (p = 0.024, 0.001 and 0.009, respectively) (Table 5). At age 38, 19.6% of women and 11.9% of men who did not report an infection were seropositive (Tables 4 and 5). At age 38, among seropositive individuals 63.3% (95% CI 54.4–71.4%) of women and 83.1% (95% CI 71.5–90.5%) of men gave no report of a Ct diagnosis (Tables 4 and 5).

**Table 4:** The Relationship between Ct Pgp3 antibody positivity and self-reported Ct, number of sexual partners, number of sexual partners of those who had not reported Ct infection and age of first coitus by age 26, 32 and 38 years. Women.

	Age 26 years			Age 32 years			Age 38 years		
	Pgp3 +ve	Total	% (95% CI)	Pgp3 +ve	Total	% (95% CI)	Pgp3 +ve	Total	% (95% CI)
**Total**	99	411	24.1 (20.0–28.5)	113	432	26.2 (22.1–30.6)	120	448	26.8 (22.7–31.1)
**Self-reported Ct by this age**									
Yes	35	44	79.5 (64.7–90.2)	39	52	75.0 (61.1–86.0)	44	59	74.6 (61.6–85.0)
No	63	360	17.5 (13.7–21.8)	74	377	19.6 (15.7–24.0)	76	387	19.6 (15.8–24.0)
			p<0.001			p<0.001			p<0.001
**Number of sexual partners**									
None	1	7	14.3 (0.4–57.9)	0	1	0.0 (0.0–0.96)	0	1	0.0 (0.0–0.96)
1	3	45	6.7 (1.4–18.3)	3	36	8.3 (1.8–22.5)	2	40	5.0 (0.6–16.9)
2–4	7	84	8.3 (3.4–16.4)	3	81	3.7 (0.8–10.4)	8	84	9.5 (4.2–17.9)
5–9	18	103	17.5 (10.7–26.2)	23	106	21.7 (14.3–30.6)	20	100	20.0 (12.7–29.2)
10–19	25	92	27.2 (18.4–37.4)	33	95	34.7 (25.2–45.2)	28	98	28.6 (19.9–38.6)
20 or more	44	75	58.7 (46.7–70.0)	49	102	48.0 (38.0–58.2)	59	117	50.4 (41.0–59.8)
			p<0.001*			p<0.001*			p<0.001*
**Number of sexual partners (if no previous self-report of Ct)**									
None	0	0	–	0	0	–	0	0	–
1	2	44	4.5 (0.6–15.5)	1	34	2.9 (0.1–15.3)	1	38	2.6 (0.1–13.8)
2–4	5	82	6.1 (2.0–13.7)	3	80	3.8 (0.8–10.6)	7	83	8.4 (3.5–16.6)
5–9	15	96	15.6 (9.0–24.5)	19	97	19.6 (12.2–28.9)	12	88	13.6 (7.2–22.6)
10–19	12	76	15.8 (8.4–26.0)	19	78	24.4 (15.3–35.4)	18	84	21.4 (13.2–31.7)
20 or more	28	57	49.1 (35.6–62.7)	30	79	38.0 (27.3–49.6)	36	88	40.9 (30.5–51.9)
			p<0.001*			p<0.001*			p<0.001*
**Age at first coitus**									
< 16 years	54	133	40.6 (32.2–49.5)	59	139	42.4 (34.1–51.1)	60	142	42.3 (34.0–50.8)
16–17 years	33	158	20.8 (14.8–28.1)	36	167	21.6 (15.6–28.6)	40	175	22.9 (16.9–29.8)
> 17 years	12	120	10.0 (5.3–16.8)	18	126	14.3 (8.7–21.6)	20	131	15.3 (9.6–22.6)
			p<0.001*			p<0.001*			p<0.001*

* χ^*2*^ *test for trend*

**Table 5:** The Relationship between Ct Pgp3 antibody positivity and self-reported Ct, number of sexual partners, number of sexual partners of those who had not reported Ct infection and age of first coitus by age 26, 32 and 38 years. Men.

	Age 26 years			Age 32 years			Age 38 years		
	Pgp3 +ve	Total	% (95% CI)	Pgp3 +ve	Total	% (95% CI)	Pgp3 +ve	Total	% (95% CI)
**Total**	47	441	10.7 (7.9–13.9)	63	449	14.0 (11.0–17.6)	59	450	13.1 (10.1–16.6)
**Self-reported Ct by this age**									
Yes	6	24	25.0 (9.8–46.7)	11	33	33.3 (18.0–51.8)	10	37	27.0 (13.8–44.1)
No	41	404	10.1 (7.4–13.5)	52	412	12.6 (9.6–16.2)	49	413	11.9 (8.9–15.4)
			P = 0.024			P = 0.001			P = 0.009
**Number of sexual partners**									
None	0	9	0.0 (0.0–33.6)	0	2	0.0 (0.0–84.2)	0	1	0.0 (0.0–97.5)
1	0	35	0.0 (0.0–10.0)	1	22	4.5 (0.1–22.8)	2	27	7.4 (0.9–24.3)
2–4	5	76	6.6 (2.2–14.7)	4	55	7.3 (2.0–17.6)	5	54	9.3 (3.0–20.3)
5–9	8	100	8.0 (3.5–15.2)	9	102	8.8 (4.1–16.1)	7	87	8.0 (3.3–15.9)
10–19	13	106	12.3 (6.7–20.1)	13	96	13.5 (7.4–22.0)	10	112	8.9 (4.4–15.8)
20 or more	21	103	20.4 (13.1–29.5)	31	150	20.7 (14.5–28.0)	35	167	21.0 (15.1–27.9)
			P<0.001*			p<0.001*			p<0.001*
**Number of sexual partners (if no previous self-report of Ct)**									
None	0	0	–	0	0	–	0	1	0.0 (0.0–97.5)
1	0	35	0.0 (0.0–10.0)	1	22	4.5 (0.1–22.8)	0	24	0.0 (0.0–14.2)
2–4	5	75	6.7 (2.2–14.9)	2	53	3.8 (0.4–13.0)	4	52	7.7 (2.1–18.5)
5–9	8	98	8.2 (3.6–15.5)	8	98	8.2 (3.6–15.5)	7	82	8.5 (3.5–16.8)
10–19	13	100	13.0 (7.1–21.2)	13	91	14.3 (7.8–23.2)	9	106	8.5 (4.0–15.5)
20 or more	15	88	17.0 (9.9–26.6)	24	131	18.3 (12.1–26.0)	29	146	19.9 (13.7–27.3)
			P = 0.001*			p = 0.001*			p<0.001*
**Age at first coitus**									
< 16 years	23	116	19.8 (13.0–28.3)	27	122	22.1 (15.1–30.5)	23	121	19.0 (12.4–27.1)
16–17 years	17	153	11.1 (6.6–17.2)	19	151	12.6 (7.7–19.0)	16	149	10.7 (6.3–16.9)
> 17 years	7	172	4.1 (1.7–8.2)	17	176	9.7 (5.7–15.0)	20	180	11.1 (6.9–16.6)
			p<0.001*			P = 0.003*			P = 0.063*

* χ^*2*^ *test for trend*

The proportion of seropositive women and men increased significantly with lifetime number of sexual partners at all three ages (p<0.001) (Tables 4 and 5). Limiting this analysis to those who never reported a Ct diagnosis, again a significant trend (p = 0.001) in seropositivity was found at all ages. At all ages, seropositivity was associated with younger first coitus in both women and men (p<0.05). This association was stronger in women than in men and did not vary by age, while in men the association weakened as they aged.

**Fig 1:**
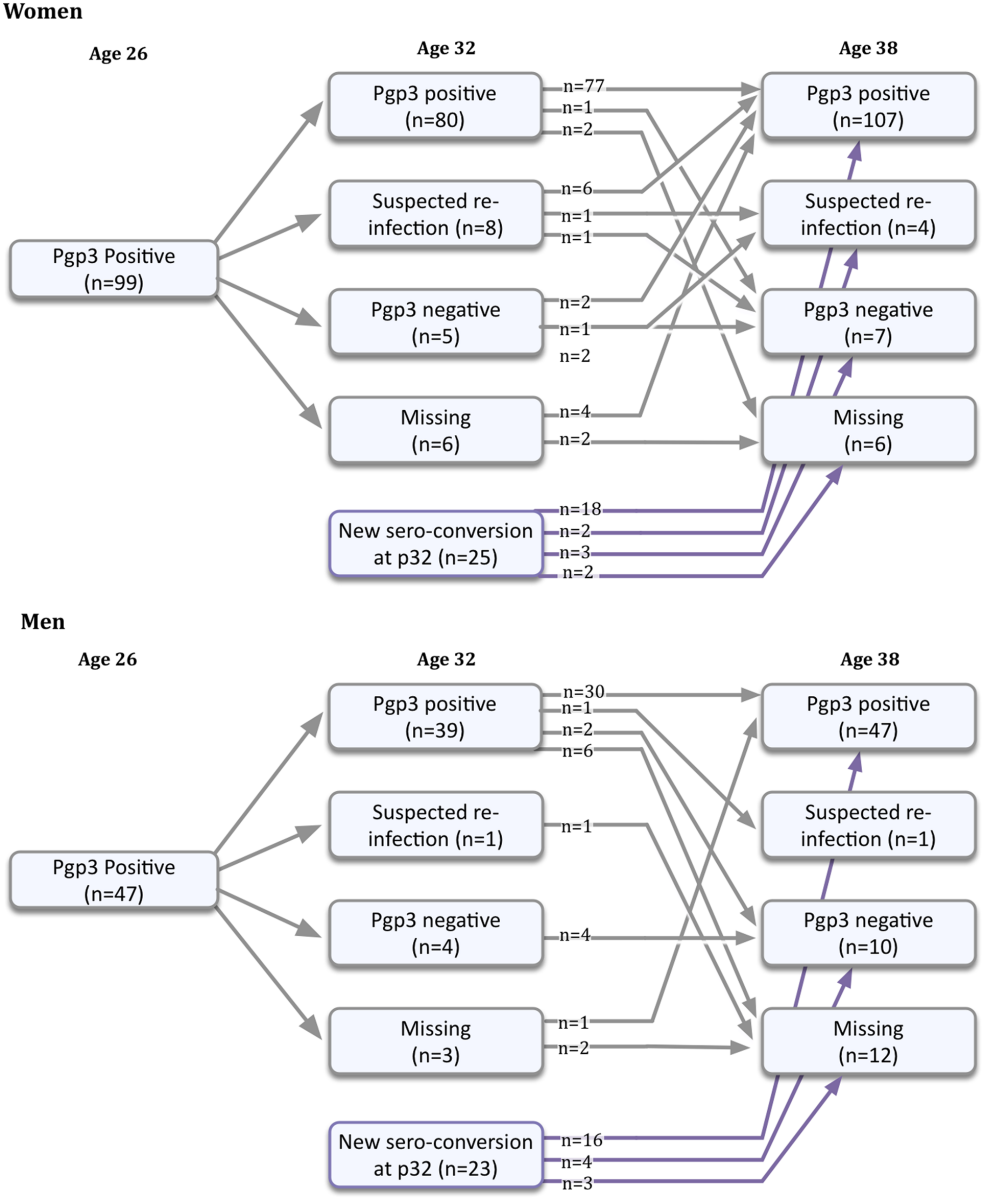
Flowchart showing the history of detectable Pgp3 antibody in women and men at age 26, 32 and 38 years. The number of women and men testing Pgp3 antibody positive at age 26 is given. Shown at age 32 and 38 are the numbers of these positive individuals who: i) maintained Pgp3 seropositivity with no self-report of re-exposure to Ct, ii) remained Pgp3 positive, but also reported a re-infection with Ct, iii) had become seronegative, and iv) were missing at that time point. Finally, the numbers of women and men who became Pgp3 seropositive between age 26 and 32 together with their subsequent antibody status and infection history by age 38 are shown.

Pgp3 antibody persisted in most women and men over the 12-year study period (Fig 1). Of the 86 women who did not report a subsequent diagnosis of Ct (suspected re-infection) retested 12 years after their first positive test at age 26, 83 (96.5% [95% CI 90.1–99.3%]) remained seropositive. This included two who were weakly positive on ELISA at age 26 and were negative at age 32. Of the 37 men retested after 12 years, 31 (83.8% [95% CI 68.0–93.8%]) remained seropositive. There was no significant difference between the percentage of men and women remaining seropositive after 12 years (p = 0.12).

## Discussion

In the epidemiological cohort, Pgp3 double-antigen ELISA positivity was associated with a history of self-reported Ct diagnosis, with the proportion seropositive higher in women self-reporting Ct than men. Seropositivity increased with increasing number of sexual partners among those with and without a history of Ct, the latter suggesting undiagnosed Ct and/or unreported Ct diagnoses. Of the seropositive women at age 26 years, 96.5% still had detectable antibody after 12 years. While the numbers were relatively small, the results suggest more persistence of antibody among women than men, and no major decline in seropositivity over this period. Similar sensitivity was observed in women attending GUM clinics and in the Dunedin cohort at age 26, but not in men making estimates of cumulative risk of Ct infection using Pgp3 serology unreliable in men.

Well-characterised sera were used to determine assay sensitivity and specificity, enabling direct comparison with the previously developed indirect Pgp3 ELISA and commercial MOMP assays. We applied the assay to a cohort from which serial sera from the same individuals were tested blind. Sexual behaviour and self-reported STIs (including Ct) were collected at each assessment [19], reducing the risk of recall bias. Serum specimens were collected and stored without incident at -80°C, so it is unlikely that antibodies would have decayed over time. The cohort population tested in this study was broadly representative of the population in New Zealand at the time of its formation, and had a very high retention at ages 26, 32 and 38 years [30]. A weakness of this sample is that the number of people with a history of infection is small, resulting in relatively wide confidence intervals around positivity rates. In addition, Ct infection in the cohort was based on self-reports which under-estimate the number of actual infections, as many will be asymptomatic and unlikely to seek testing [31, 32]. Alternatively, some who report Ct infection may not have been infected.

Although the double-antigen ELISA demonstrated higher sensitivity than the indirect ELISA, it requires a 25-fold higher volume of serum. In the initial analysis of Ct-exposed and paediatric samples, no samples with indirect assay absorbance values <0.1 were positive when assayed by the double-antigen ELISA, nor did any with values >1.0 test negative. Further analyses of the Dunedin samples by the indirect ELISA (Table D in S1 Text) indicate that in large-scale studies the indirect ELISA is suitable for initial screening, and subsequent testing (sera with absorbance values between 0.1 and 1.0) by the double-antigen ELISA.

Public Health intervention strategies against Ct have been implicated in the rising number of infections on the basis that they undermine the development of protective immunity [33, 34]. Our findings lead us to the conclusion that, if arrested immunity occurs following early treatment, it does not substantially affect Pgp3 antibody development and persistence in women [6, 33, 34]. At age 26 years we observed in women a similar high detection of Pgp3 antibody (~80%) in Ct detection-positive GUM attendees, who are more likely to be incident cases [35], and the Dunedin cohort who are possibly more likely to be prevalent cases. Thus Pgp3 antibody could be used to estimate cumulative risk of infection in female populations in which control programmes are taking place [6, 36]. In an accompanying paper, Woodhall *et al*. use data and stored sera from nationally-representative household surveys from 1994 to 2012 in England to evaluate, using Pgp3 antibody the impact of widespread opportunistic Ct screening [36].

The higher proportion of women who are Pgp3 seropositive also holds true for MOMP peptide serology [9, 21, 37]. The mechanism for this is unclear, and merits further investigation [9, 21]. We observed a lower Pgp3 sensitivity, 25%, in men in the Dunedin cohort than the 54.4% observed in Ct detection-positive men attending GUM clinics. We believe the most likely explanation is poor diagnostic test accuracy in the 1990s resulting in some men in the Dunedin cohort being mis-diagnosed with Ct and/or recall bias [38]. If we consider only asymptomatic men with no history of infection attending GUM clinics, probably many with incident infection [35], the observed 25% sensitivity remains lower than the 43% observed in this GUM population. This low sensitivity cannot be explained by the hypothesis that early Ct treatment stops the development of Pgp3 antibody, as this would be more likely to affect the asymptomatic men attending GUM clinics than those in the Dunedin cohort [33, 35].

The clear increase of Pgp3 positivity with increasing number of sexual partners, a major risk factor for Ct and other STIs [39, 40], shows Pgp3 antibody can be used as a marker of Ct risk, especially among women. That this correlation persisted when restricted to those who did not report Ct is strong evidence for under-diagnosis and/or reporting in epidemiological studies, although not all seropositive results will be due to Ct infection as the specificity of the test is around 98%.

We observed that 63.3% women and 83.1% men who were seropositive at age 38 years did not report having ever been diagnosed with Ct, while around 2% may be false positives, this indicates that the majority of Ct infection was undetected/unreported in this cohort. Furthermore, there was an association of seropositivity with younger age of first sexual intercourse. This probably reflects a greater cumulative risk of Ct infection [41], but could also be accentuated by the possible increased biological susceptibility to Ct infection in younger women [42].

A key feature of this study was the repeated serological testing over a 12-year period. Among women reporting any past infection, 79.5% at age 26, 75% at age 32 and 74.6% at age 38 were antibody positive. While we have no information on when individuals became infected and could not examine the proportion of those who had seroconverted at some point in the past with subsequent seroreversion [7], we demonstrate antibody persistence over a 12-year period of 96.5% for women and 83.8% for men. While we excluded those reporting a subsequent Ct infection, we could not rule out unrecognised and/or reported reinfection that could explain the two women who were initially seropositive, negative six years later, then again positive 12 years later. The finding of persistence is consistent with previous analyses in which Pgp3 positivity in women was high soon after infection and remained stable at around 64% after a year [7]. While the point estimates suggest lower persistence for men, our numbers precluded examining this usefully. We are currently quantifying the loss of antibody since time of treatment in women attending departments of sexual health. This information will be used to develop the methodology in order to obtain estimates of Ct incidence and prevalence in England using serial population-based serum collections.

## Conclusion

We show Ct infection was both common and usually undetected in women in this New Zealand cohort born in 1972 and 1973, with almost a third of women by this age having been infected by age 38, and demonstrate a strong and consistent correlation of Pgp3 seropositivity with self-reported Ct, multiple sexual partners and age of first intercourse. We conclude that our simple indirect assay facilitates high through-put antibody screening, and equivocal specimens can then be reconciled by the double-antigen ELISA.

Our findings suggest that surveys could make use of Pgp3 antibody in different age groups to examine the temporal change in cumulative risk of infection particularly among women, although persistence beyond 12 years has not yet been investigated [36]. These data provide a powerful argument for a role for Ct Pgp3 serology in evaluating Ct control programmes [36].

## Supporting Information

S1 Text Additional tables and text comparing the performance of the indirect and double-antigen ELISAs. (DOCX)
