# Supplementary material for: Chlamydia trachomatis Pgp3 Antibody Persists and Correlates with Self-Reported Infection and Behavioural Risks in a Blinded Cohort Study
Source: PLoS One. 2016 Mar 14;11(3):e0151497. doi: 10.1371/journal.pone.0151497 (PMC4790965; doi:10.1371/journal.pone.0151497)
Supplement: S1 Text — (DOCX) [file pone.0151497.s001.docx]

**Table A. All pairwise comparison in sensitivity of Ct antibody assays - female GUM clinic samples**

| **Assay** | **Pgp3 double antigen** | | | **Pgp3 indirect** | | | **Anilabsystems** | | | **SeroCT** | | | **Medac** | | |
| --- | --- | --- | --- | --- | --- | --- | --- | --- | --- | --- | --- | --- | --- | --- | --- |
|  | **Mean**  **Difference** | **95% CI** | | **Mean**  **difference** | **95% CI** | | **Mean**  **difference** | **95% CI** | | **Mean**  **difference** | **95% CI** | | **Mean**  **difference** | **95% CI** | |
| **Pairwise comparisons** | |  |  |  |  |  |  |  |  |  |  |  |  |  |  |
| **Pgp3 double antigen** | |  |  |  |  |  |  |  |  |  |  |  |  |  |  |
| **Pgp3 indirect** | -11.4 | [-17.9 | -4.9] |  |  |  |  |  |  |  |  |  |  |  |  |
| **Anilabsystems** | -23.4 | [-30.4 | -16.5] | -12.0 | [-19.4 | -4.7] |  |  |  |  |  |  |  |  |  |
| **SeroCT** | -27.8 | [-34.9 | -20.8] | -16.5 | [-23.9 | -9.0] | -4.4 | [-12.1 | 3.3] |  |  |  |  |  |  |
| **Medac** | -36.7 | [-43.7 | -29.7] | -25.3 | [-32.8 | -17.9] | -13.3 | [-21.0 | -5.6] | -8.9 | [-16.6 | -1.1] |  |  |  |
| **MIF** | -19.0 | [-25.8 | -12.2] | -7.6 | [-14.9 | -0.3] | 4.4 | [-3.2 | 12.0] | 8.9 | [1.2 | 16.5] | 17.7 | [10.1 | 25.4] |
| **Pairwise comparisons adjusted for multiple comparisons** | | | | | |  |  |  |  |  |  |  |  |  |  |
| **Pgp3 double antigen** | |  |  |  |  |  |  |  |  |  |  |  |  |  |  |
| **Pgp3 indirect** | -11.4 | [-22.5 | -0.3] |  |  |  |  |  |  |  |  |  |  |  |  |
| **Anilabsystems** | -23.4 | [-35.2 | -11.6] | -12.0 | [-24.5 | 0.5] |  |  |  |  |  |  |  |  |  |
| **SeroCT** | -27.8 | [-39.8 | -15.9] | -16.5 | [-29.1 | -3.8] | -4.4 | [-17.5 | 8.7] |  |  |  |  |  |  |
| **Medac** | -36.7 | [-48.6 | -24.8] | -25.3 | [-37.9 | -12.7] | -13.3 | [-26.4 | -0.2] | -8.9 | [-22.1 | 4.3] |  |  |  |
| **MIF** | -19.0 | [-30.6 | -7.4] | -7.6 | [-20.0 | 4.8] | 4.4 | [-8.4 | 17.3] | 8.9 | [-4.1 | 21.8] | 17.7 | [4.7 | 30.7] |

**Table B. All pairwise comparison in sensitivity of Ct antibody assays - male GUM clinic samples**

| **Assay** | **Pgp3 double antigen** | | | **Pgp3 indirect** | | | **Anilabsystems** | | | **SeroCT** | | | **Medac** | | |
| --- | --- | --- | --- | --- | --- | --- | --- | --- | --- | --- | --- | --- | --- | --- | --- |
|  | **Mean**  **difference** | **95% CI** | | **Mean**  **difference** | **95% CI** | | **Mean**  **difference** | **95% CI** | | **Mean**  **difference** | **95% CI** | | **Mean**  **difference** | **95% CI** | |
| **Pairwise comparisons** | |  |  |  |  |  |  |  |  |  |  |  |  |  |  |
| **Pgp3 double antigen** | |  |  |  |  |  |  |  |  |  |  |  |  |  |  |
| **Pgp3 indirect** | -7.1 | [-14.8 | 0.5] |  |  |  |  |  |  |  |  |  |  |  |  |
| **Anilabsystems** | -14.2 | [-21.8 | -6.6] | -7.0 | [-14.7 | 0.6] |  |  |  |  |  |  |  |  |  |
| **SeroCT** | -14.8 | [-22.4 | -7.2] | -7.7 | [-15.3 | -0.1] | -0.7 | [-8.2 | 6.9] |  |  |  |  |  |  |
| **Medac** | -12.1 | [-19.7 | -4.5] | -4.9 | [-12.6 | 2.7] | 2.1 | [-5.5 | 9.7] | 2.7 | [-4.8 | 10.3] |  |  |  |
| **MIF** | -6.0 | [-13.7 | 1.6] | 1.1 | [-6.6 | 8.8] | 8.1 | [0.5 | 15.8] | 8.8 | [1.2 | 16.4] | 6.0 | [-1.6 | 13.7] |
| **Pairwise comparisons adjusted for multiple comparisons** | | | | | |  |  |  |  |  |  |  |  |  |  |
| **Pgp3 double antigen** | |  |  |  |  |  |  |  |  |  |  |  |  |  |  |
| **Pgp3 indirect** | -7.1 | [-20.2 | 5.9] |  |  |  |  |  |  |  |  |  |  |  |  |
| **Anilabsystems** | -14.2 | [-27.1 | -1.3] | -7.0 | [-20.0 | 5.9] |  |  |  |  |  |  |  |  |  |
| **SeroCT** | -14.8 | [-27.7 | -2.0] | -7.7 | [-20.6 | 5.2] | -0.7 | [-13.5 | 12.1] |  |  |  |  |  |  |
| **Medac** | -12.1 | [-25.0 | 0.9] | -4.9 | [-17.9 | 8.0] | 2.1 | [-10.8 | 15.0] | 2.7 | [-10.1 | 15.6] |  |  |  |
| **MIF** | -6.0 | [-19.1 | 7.0] | 1.1 | [-11.9 | 14.1] | 8.1 | [-4.8 | 21.1] | 8.8 | [-4.1 | 21.7] | 6.0 | [-6.9 | 19.0] |

**Table C. All pairwise comparison in specificity of Ct antibody assays - paediatric samples excluding the 11 MIF+ samples**

| **Assay** | **Pgp3 double antigen** | | | | **Pgp3 indirect** | | | **Anilabsystems** | | | **SeroCT** | | |
| --- | --- | --- | --- | --- | --- | --- | --- | --- | --- | --- | --- | --- | --- |
|  | **Mean**  **difference** | **95% CI** | | | **Mean**  **difference** | **95% CI** | | **Mean**  **difference** | **95% CI** | | **Mean**  **difference** | **95% CI** | |
| **Pairwise comparisons** | |  |  | |  |  |  |  |  |  |  |  |  |
| **Pgp3 double antigen** | |  |  | |  |  |  |  |  |  |  |  |  |
| **Pgp3 indirect** | 0.0 | [-1.7 | 1.7] | |  |  |  |  |  |  |  |  |  |
| **Anilabsystems** | 1.2 | [-0.3 | 2.7] | | 1.2 | [-0.3 | 2.7] |  |  |  |  |  |  |
| **SeroCT** | -0.8 | [-2.7 | 1.1] | | -0.8 | [-2.7 | 1.1] | -2.0 | [-3.7 | -0.4] |  |  |  |
| **Medac** | -3.0 | [-5.3 | -0.8] | | -3.0 | [-5.3 | -0.8] | -4.3 | [-6.3 | -2.2] | -2.2 | [-4.6 | 0.1] |
| **Pairwise comparisons adjusted for multiple comparisons** | | | | | | |  |  |  |  |  |  |  |
| **Pgp3 double antigen** | |  |  |  | |  |  |  |  |  |  |  |  |
| **Pgp3 indirect** | 0.0 | [-2.7 | 2.7] |  | |  |  |  |  |  |  |  |  |
| **Anilabsystems** | 1.2 | [-1.1 | 3.6] | 1.2 | | [-1.1 | 3.6] |  |  |  |  |  |  |
| **SeroCT** | -0.8 | [-3.8 | 2.2] | -0.8 | | [-3.8 | 2.2] | -2.0 | [-4.6 | 0.6] |  |  |  |
| **Medac** | -3.0 | [-6.6 | 0.5] | -3.0 | | [-6.6 | 0.5] | -4.3 | [-7.5 | -1.0] | -2.2 | [-5.9 | 1.5] |

**Table D. Comparison of indirect and double-antigen ELISAs in the Dunedin cohort by absorbance values on indirect assay**

|  | | **Double-antigen sandwich ELISA (absorbance value (450–620nm) range)** | |
| --- | --- | --- | --- |
|  |  | **Negative (<0.44^2^)** | **Positive(≥0.44^2^)** |
| **Indirect ELISA (Absorbance value (450-620nm) range )** | **Negative (<0.1)** | 1817 | 10 |
|  | **Negative (0.1–0.473^1^)** | 305 | 233 |
|  | **Positive (0.473^1^–1.0)** | 12 | 96 |
|  | **Positive (>1.0)** | 4 | 164 |

^1^An absorbance (450-620nm) value of 0.473 is the cutoff for the indirect assay and ^2^0.44 the cutoff for the double-antigen assay

Results of testing Dunedin cohort by both the indirect and double-antigen ELISA were assessed to develop a strategy for testing larger numbers of samples. The indirect ELISA can be used for screening serum samples. Those samples giving absorbance values between 0.1-1.0 are retested by the double-antigen ELISA, which requires a 25-fold larger volume of serum. Samples are then considered seropositive if they are: 1) above the cut-off value for re-testing (absorbance value >1.0 on the indirect ELISA), 2) positive by the double-antigen ELISA. Samples are considered seronegative if they are: 1) below the cut-off value for re-testing by the indirect (absorbance value <0.1 on the indirect ELISA), 2) were positive on the indirect ELISA (absorbance value >0.473) but negative when retested by the double-antigen ELISA. This testing strategy represents a 98.0% sensitivity, and 99.8% specificity compared to the results that would have been achieved had all samples been tested on the double-antigen ELISA. Ten of the samples tested were not included in the subsequent analysis due to lack of behavioural data (Tables 4 and 5).
